# The Role of TLR2 and TLR4 in Recognition and Uptake of the Apicomplexan Parasite *Eimeria bovis* and Their Effects on NET Formation

**DOI:** 10.3390/pathogens10020118

**Published:** 2021-01-24

**Authors:** Tamara Muñoz-Caro, Amanda J. Gibson, Iván Conejeros, Dirk Werling, Anja Taubert, Carlos Hermosilla

**Affiliations:** 1 Institute of Parasitology, Justus Liebig University Giessen, 35392 Giessen, Germany; tmunoz6@santotomas.cl (T.M.-C.); ivan.conejeros@vetmed.uni-giessen.de (I.C.); Anja.Taubert@vetmed.uni-giessen.de (A.T.); 2 Escuela de Medicina Veterinaria, Facultad de Recursos Naturales y Medicina Veterinaria, Universidad Santo Tomás, Talca 3460000, Chile; 3 Department of Pathobiology and Population Sciences, Royal Veterinary College, Hatfield AL9 7TA, UK; amg39@aber.ac.uk (A.J.G.); Dwerling@rvc.ac.uk (D.W.); 4 Centre of Excellence in Bovine Tuberculosis, Institute for Biological, Environmental and Rural Sciences, Aberystwyth University, Wales SY23 3FD, UK

**Keywords:** *E. bovis*, TLR, IL-8, neutrophil extracellular traps

## Abstract

Background: Bovine polymorphonuclear neutrophils (PMN) constitutively express the Toll-like receptors (TLRs) TLR2 and TLR4 and have been shown to generate Neutrophil extracellular traps (NETs) upon exposure to *Eimeria bovis*. The present work investigated the role of TLR2 and TLR4 in the recognition and uptake of *E. bovis* sporozoites, IL-8 production and neutrophil extracellular trap (NET) formation. Methods: TLR expression was performed by flow cytometric analysis on PMN exposed to live carboxyfluorescein succinimidyl ester (CFSE)-stained sporozoites. Supernatants of PMN exposed to different *E. bovis* sporozoite preparations and antigens in the absence or presence of TLR antibodies were assessed for IL-8 secretion. Cells were exposed to sporozoite preparations and assessed for the activation of transcription factor NF-κB using a luciferase reporter assay. Immunofluorescence analysis was done to investigate TLR2 and TLR4 surface expression and NET formation on bovine PMN exposed to vital sporozoites. Results: we observed significantly increased TLR2 and TLR4 expression with a mean increase in expression that was greater for TLR2 than TLR4. This upregulation neither inhibited nor promoted sporozoite phagocytosis by bovine PMN. Live sporozoites together with anti-TLR2 mAb resulted in a significant enhancement of IL-8 production. NF-κB activation was more strongly induced in TLR2-HEK cells than in TLR4/MD2-HEK cells exposed to heat-killed sporozoites and antigens. Immunofluorescence analysis showed TLR-positive signals on the surface of PMN and concomitant NET formation. Conclusions: This is the first report on *E. bovis*-induced concomitant TLR2 and TLR4 expression during bovine PMN-derived NETosis.

## 1. Introduction

At least thirteen monoxenous apicomplexan *Eimeria* species have been reported to infect domestic cattle worldwide to date and, among these species, *E. bovis* is considered as one of the most pathogenic species, causing severe inflammation of the intestine with clinical manifestations such as haemorrhagic diarrhoea, dehydration, weight loss and poor growth rates, mainly affecting calves [1,2]. Underlying *E. bovis* infections are complex host adaptive [3,4,5] as well as host innate immunological regulation in vitro [6,7,8], ex vivo [9] and in vivo [10]; however, little is known about pathogen recognition receptors (PRRs) involved in early innate immune reactions against ruminant *Eimeria* species.

Polymorphonuclear neutrophils (PMN) are considered the first line of defence of the early host innate immune response [11,12] and constitutively express PRRs, including Toll-like receptors (TLRs), dectin-1 and CD11b on their surface [13,14,15,16] as well as cytosolic PRRs recognizing pathogen-associated molecular patterns such as retinoid acid-inducible gene-I (RIG-I)-like receptors (RLRs) and NODs [17]. Key PMN-derived defence mechanisms have been classically defined as a variety of potent intracellular/extracellular microbicidal mechanisms to efficiently kill invasive pathogens, such as bacteria, viruses, fungi [18,19] and large protozoan and helminth parasites [20,21,22] and to stimulate adaptive defence mechanisms [23,24,25,26,27]. PMN-derived effector mechanisms include phagocytosis, reactive oxygen species (ROS) production, secretion of granules containing several antimicrobial proteins [24,28], casting of neutrophil extracellular traps (NETs) [29,30] and chemokine/cytokine production, thereby inducing the arrival of other leukocytes to the site of infection or inflammation [31,32]. 

TLRs sense pathogen-associated molecular patterns (PAMPs) such as microbial membrane components, including lipoproteins (ligands for TLR2), lipopolysaccharide (LPS; ligand for TLR4), flagellin and nucleic acids of bacterial and parasitic origin [33,34,35]. The presence of TLRs in host innate immune leukocytes permits an initial response which is subsequently amplified by the host adaptive immune system [36]. In contrast to other PRRs, such as C-type lectin receptors, for example, the binding of a ligand to its TLR seems to impact more on the subsequent signaling event, rather than increasing phagocytosis. TLR-induced intracellular signaling pathways can be broadly classified as MyD88-dependent, MyD88-independent or TRIF-dependent pathways. Three major signaling pathways are responsible for mediating TLR-induced responses: (i) NF-κB, (ii) mitogen-activated protein kinases (MAPKs) and (iii) IFN regulatory factors (IRFs) [37,38]. NF-κB and MAPK signaling pathways play central roles in the induction of proinflammatory responses, the upregulation of maturation molecules (e.g., MHC II) and the transcription and secretion of IL-1, IL-6, IL-12 and TNF-α [39].

Understanding the role of TLR activation and evidence for specific responses to parasite-derived molecules are growing, particularly in the field of apicomplexan protozoans. It has been consistently demonstrated that MyD88, TLR2, TLR9 and, to a smaller extent, TLR4 play a significant role in the activation of host innate immune response against *Plasmodium falciparum* [33,34,40,41]. In addition, it has been shown that glycophosphatidylinositol (GPI) of *Toxoplasma gondii* is recognized by TLR2 and TLR4 [42], together with the ability of murine TLR11 and TLR12 to bind to *T. gondii*-associated profilin-like proteins [43,44,45]. Besides, it has been demonstrated that a complex of TLR2–TLR6 and CD14 are involved in the recognition of *Trypanosoma cruzi*-derived molecules [35]. In line with this, *T. cruzi*-soluble antigens are able to induce NET release by stimulating TLR2 and TLR4 on exposed PMN [46]. Some other protozoan-specific molecules, such as lipophosphoglycans (LPGs) of the closely related euglenozoan parasite *Leishmania major*, have been shown to interact with TLR2, and further to decrease TLR9 expression in peritoneal macrophages, resulting in reduced anti-leishmanicidal responses in murine BALB/c models [47]. Conversely, the role of PMN-expressed TLRs against neglected monoxenous ruminant *Eimeria* species has been investigated to a lesser extent to date. 

Of particular interest is *E. bovis*, for which endogenous sporozoites develop within highly reactive endothelial host cells (i.e., host cells of the innate immune system) of the small intestine thereby forming huge macromeronts of 300 µm in size and producing > 170,000 stage I merozoites, which then undergo a second merogony and finally a sexual gamogony in epithelial host cells of large intestine [2,48]. This massive intracellular *E. bovis* replication can lead to severe inflammation of the intestine, resulting in PMN-derived effector mechanisms. Indeed, bovine PMN have previously been shown to be important in early host innate immune responses against *E. bovis* in vitro [49,50], as well as ex vivo [51], thereby interacting directly not only with parasites but also with extracted parasite-specific antigens [6,50]. We have also shown that besides PMN-derived classical effector mechanisms, bovine PMN cast extensive NETs in response to *E. bovis* sporozoites and oocysts in vitro and in vivo [6,7,10], suggesting specific interactions of PMN with different *E. bovis* stages (e.g., sporozoites, merozoites, sporocysts, oocysts). All these parasite stages have different antigens in order to overcome adverse early host innate immune reactions, and the recognition of *E. bovis*-derived PAMPs might be mediated by bovine TLRs. More importantly, bovine PMN have been described as expressing numerous PRRs [13,14]. In cattle, TLRs have been described on a number of innate immune cells and are associated with the recognition of *Mycobacterium tuberculosis* and *M. bovis* by macrophages [52], pathogens involved in bovine respiratory disease [53] and *Escherichia coli*-mediated mastitis [54]. Bovine PMN express TLR2 and TLR4 and the function of TLR2 has been demonstrated by stimulation with Pam_3_CSK_4_, inducing a proinflammatory response [16,55]. 

To date, few data exist showing the activation of TLRs in leukocytes of the bovine innate immune system in response to parasite-derived molecules, with only one report assessing the potential involvement of PMN receptors in early innate immune responses against *E. bovis* [7]. As such, *E. bovis*-induced NETosis was shown to be mediated by CD11b expressed on parasite-exposed bovine PMN [7]. We therefore sought to explore the possible role of TLR2 and TLR4 not only in the uptake of *E. bovis* sporozoites but also in PMN-derived pro-inflammatory reactions as well as NET extrusion. 

## 2. Results

### 2.1. Addition of TLR2/4 Antibodies Does Not Seem to Inhibit Phagocytosis of E. bovis by PMN, but Seems to Stabilize Their Surface Expression

**Figure 1:**
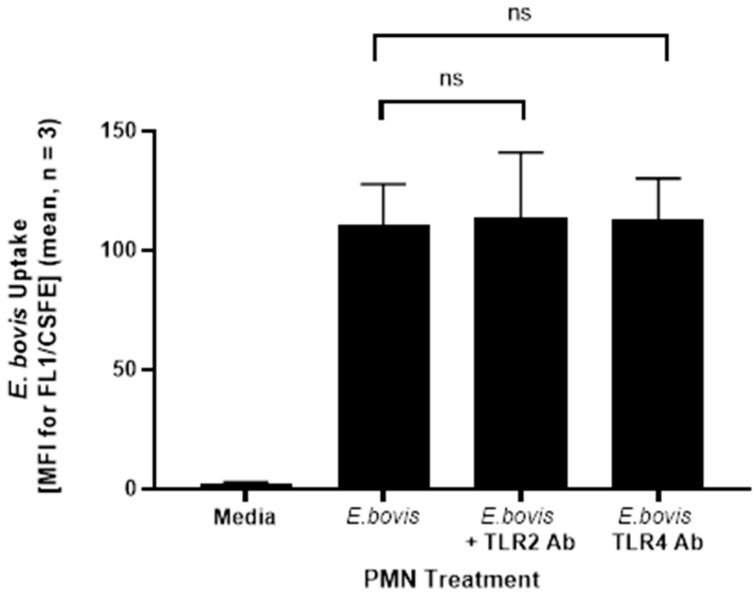
Pre-incubation with directly labeled TLR2 or TLR4 antibodies does not impact on phagocytosis of carboxyfluorescein succinimidyl ester (CSFE)-labeled *E. bovis* by polymorphonuclear neutrophils (PMN). Isolated PMN (1 × 10^6^ per sample; *n* = 3) were pre-treated with TLR2 and TLR4 antibodies for 30 min and exposed to carboxyfluorescein succinimidyl ester (CFSE)-labeled *E. bovis* sporozoites (2.5 µm, 30 min) at a 1:1 ratio for two hours for subsequent flow cytometric analysis. Pre-incubation of PMN with antibodies to bovine TLR2 and TLR4 did not seem to impact on the phagocytosis of CFSE-labeled *E. bovis* sporozoites. Data are represented as the mean of 3 replicates ± SD and were analyzed using an unpaired Student’s *t*-test using GraphPad Prism V.8.4.3 (GraphPad Software Inc., San Diego, CA, USA).

In the first set of experiments, we tried to assess the impact of blocking TLR2 and TLR4 with corresponding directly labeled antibodies on the uptake of carboxyfluorescein succinimidyl ester (CSFE)-labeled *E. bovis* by PMN. PMN isolated from three animals were incubated with antibodies to TLR2 and TLR4 for 30 min prior to exposure to live *E. bovis* for two hours. However, neither antibody had an effect of *E. bovis* phagocytosis by PMN compared to *E. bovis* in media alone (Figure 1).

**Figure 2:**
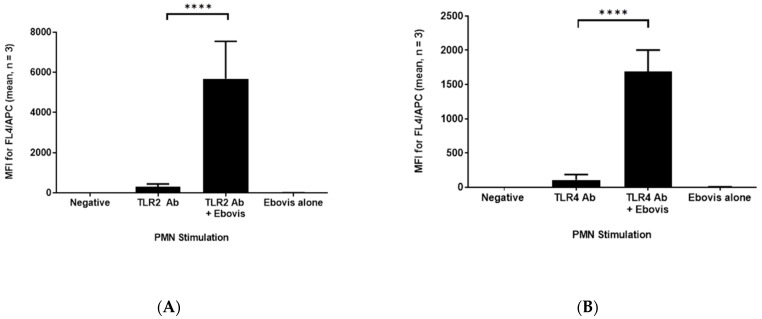
*E. bovis* increases TLR2 and TLR4 expression on PMN. PMN (1 × 10^6^ per sample; *n* = 3) were incubated with TLR2 and TLR4 antibodies for 30 min prior to exposure to live *E. bovis* for two hours for subsequent Flow cytometry analyses (FACS). Incubation of PMN with *E. bovis* significantly increases TLR2 (**A**) and TLR4 (**B**) expression (**** *p* < 0.0001). Data are represented as the mean of 3 replicates ± SD and were analyzed using an unpaired Student’s *t*-test using GraphPad Prism V.8.4.3 (GraphPad Software Inc.). *p*-value notation; * *p* < 0.05, ** *p* < 0.01, *** *p* < 0.001, **** *p* < 0.0001.

Interestingly, though, we observed a repeated effect on the staining of TLR2 and TLR4 using the same directly labeled antibodies. In the absence of *E. bovis*, there was a relatively low surface expression of both TLRs, and neither of the directly labeled antibodies bound to CSFE-labeled *E. bovis* on its own. However, incubation of PMN with CSFE-labeled *E. bovis* seemed to significantly increase the detection of both TLR2 and TLR4 on the cellular surface (Figure 2A,B, respectively, *p* < 0.0001). 

### 2.2. Exposure of Bovine PMN to E. bovis Increases IL-8 Secretion in the Presence of TLR2 Antibodies

**Figure 3:**
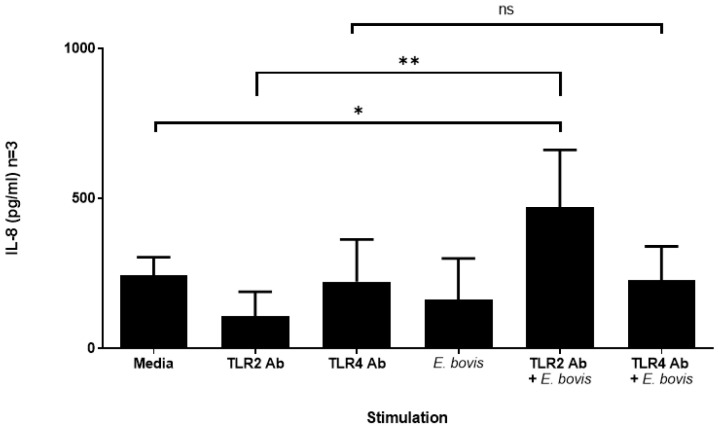
IL-8 production in PMN upon *E. bovis* exposure. Supernatants of PMN (1 × 10^6^ per sample; *n* = 3) treated with TLR2 and TLR4 antibodies and exposed to live *E. bovis* sporozoites (1:1 ratio; 2 h) were assessed for the presence of IL-8 by ELISA analysis. TLR2-treated PMN exposed to sporozoites showed a significant increase in IL-8 production (* *p* < 0.05) when compared to PMN in media. Likewise, a significant increase in IL-8 production (** *p* < 0.01) was observed in the same experimental condition when compared to the respective control without exposure to *E. bovis.* Data are represented as the mean of 3 replicates ± SD and were analyzed using an unpaired Student’s *t*-test using GraphPad Prism V.8.4.3 (GraphPad Software Inc., San Diego, CA, USA). *p*-value notation; * *p* < 0.05, ** *p* < 0.01, *** *p* < 0.001, **** *p* < 0.0001.

TLRs have been suggested to be mainly involved in inducing cell signaling events, rather than inducing phagocytosis [56,57]. Thus, having established that the exposure of bovine PMN to *E. bovis* induces the expression of TLR2 and TLR4 without impacting uptake, we next assessed whether enhanced expression is concomitant with an increased secretion of IL-8, one of the key chemokines secreted by activated PMN. Supernatants of PMN exposed to *E. bovis* (with and without TLR antibodies) were assessed for the presence of IL-8 by ELISA analysis. Despite increases in the surface expression of both TLRs, although greater for TLR2 than TLR4, in PMN exposed to *E. bovis*, only sporozoites together with anti-TLR2 mAb resulted in enhanced IL-8 production (Figure 3, *p* < 0.05 when compared to media alone). Furthermore, antibodies alone did not induce significant IL-8 responses, indicating that the increased TLR2 expression observed (Figure 1) is functional when exposed to *E. bovis* antigen (*Eb*Ag). 

### 2.3. Induction of TLR2 and TLR4 Activation by E. bovis Sporozoites 

**Figure 4:**
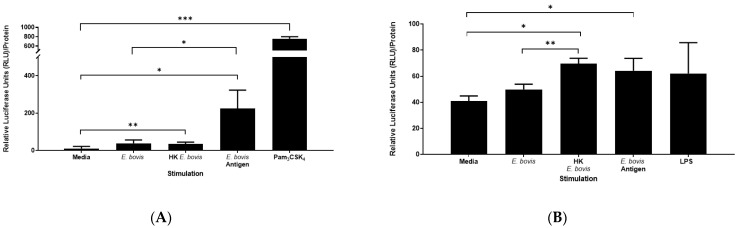
Induction of Toll-like receptor (TLR)-dependent NF-κB activation by *E. bovis* sporozoites. In order to investigate the activation of TLRs in bovine PMN, we used HEK cells expressing either bovine TLR2 (A) or a combination of bovine TLR4/MD2 (B). Cells were exposed to different *E. bovis* sporozoite preparations: live, heat killed (HK) or antigen (*Eb*Ag) for 24 h and assessed for activation of transcription factor NF-κB using a luciferase reporter assay. (**A**) HK sporozoites and *Eb*Ag induced substantial TLR2-dependent NF-κB activation compared to media alone (*** *p* < 0.0001, ** *p* < 0.01 and * *p* < 0.05, respectively). TLR2-induced NF-κB significantly increases when exposed to *Eb*Ag compared to live *E. bovis* (*p* < 0.05). (**B**) HK sporozoites induced a significant NF-κB response when compared to media (*p* < 0.05) and when compared to live parasitic stages (*p* < 0.01). A significant increase in NF-κB response was observed in *Eb*Ag when compared to media (*p* < 0.05). In both experiments, Pam_3_CSK_4_ and Lipopolysaccharides (LPS) served as ligand controls for TLR2 and TLR4/MD2, respectively, and phorbol 12-myristate 13-acetate (PMA) was used as an NF-κB technical control (data not shown for clarity). Data are represented as the mean of 3 replicates ± SD and were analyzed using an unpaired Student’s *t*-test using GraphPad Prism V.8.4.3 (GraphPad Software Inc.).

To assess whether the response seen in *E. bovis*-exposed bovine PMN was indeed due to an interaction and activation of TLRs expressed by PMN, we aimed to confirm this activation using HEK cells expressing either bovine TLR2 or a combination of bovine TLR4/MD2. Cells were exposed to live *E. bovis* sporozoites, heat-killed (HK) *E. bovis* sporozoites or *Eb*Ag for 24 h and assessed for the activation of transcription factor NF-κB using a luciferase reporter assay (Figure 4A,B). Pam_3_CSK_4_ and lipopolysaccharides (LPS) served as ligand controls for TLR2 and TLR4/MD2, respectively, and phorbol 12-myristate 13-acetate (PMA) was used as an NF-κB technical control. 

Both HEK-TLR2 and HEK-TLR4 cells responded to assay control stimulation PMA (mean = 441.82 RFU and 411.37 RFU, respectively, data not shown). Specifically, in TLR2-expressing HEK cells, Pam_3_CSK_4_ induced the strongest relative luciferase unit (RFU) response. Interestingly, of the *E. bovis* preparations used, only HK sporozoites and soluble *Eb*Ag induced substantial TLR2-dependent NF-κB activation compared to media alone (*p* < 0.01 and *p* < 0.05, respectively; Figure 4A). Intact (live) *E. bovis* sporozoites induced less NF-κB activation than HK *E. bovis* sporozoites, but still above the media control (Figure 4A). TLR2-induced NF-κB significantly increases when exposed to *Eb*Ag compared to live sporozoites of *E. bovis* (*p* < 0.05).

In addition, TLR4 induction of NF-κB shows a similar pattern (Figure 4B), with HK *E. bovis* sporozoites inducing a stronger NF-κB response (*p* < 0.01) compared to live parasitic stages and (*p* < 0.05) when compared to media. Similarly, a significant increase in NF-κB response was observed for soluble *Eb*Ag when compared to media alone (*p* < 0.05). In agreement with IL-8 secretion by PMN, the magnitude of TLR2-induced NF-κB signaling is significantly greater than for TLR4. 

### 2.4. E. bovis-Induced TLR2 and TLR4 Activation Resulted in NETosis of Exposed Bovine PMN

**Figure 5:**
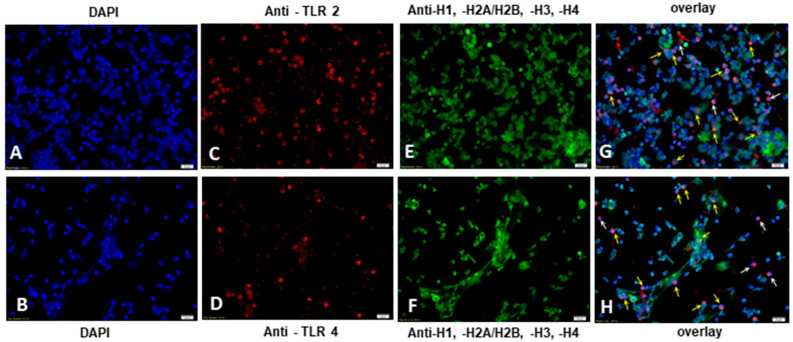
Immunofluorescence analysis on bovine PMN activation of TLR2 and TLR 4 by *E. bovis* and concomitant neutrophil extracellular trap (NET) formation. PMN (*n* = 3; 5 × 10^5^) were exposed to vital *E. bovis* sporozoites (ratio 1:1) on poly-l-lysine-treated coverslips (120 min, 37 °C) and fixed for further antibody exposure (60 min) with anti-TLR2 (**C**) and anti-TLR4 (**D**) antibodies and anti-histone H1, H2A/H2B, H3, H4 antibody (**E**,**F**). Coverslips were mounted with ProLong Antifade containing DAPI (**A**,**B**) which was used for observation of PMN nuclei and NET extracellular DNA by fluorescence microscopy analysis. In both cases, expression of TLR2 and TLR4 was observed on the surface of bovine PMN (red) co-localized with NET-derived histones (green) and extracellular DNA (blue), as indicated by yellow arrows ((**G**,**H**)—overlay of images collected for nucleic acid, TLR2/TLR and histone staining). Co-localization of TLR-positive signals with early stages of NETosis are indicated by white arrows (**G**,**H**). TLR-positive signals without NETosis are indicated by orange arrows (**G**,**H**). Images were visualized by using an inverted Olympus IX81^®^ epifluorescence microscope equipped with a digital camera (XM10^®^, Olympus, Tokyo, Japan). Scale bar magnitude: 20 µm.

We and others have demonstrated previously that *E. bovis* is able to strongly induce NETosis in bovine PMN in vitro and in vivo [6,7,10]. Therefore, based on the present results, we investigated whether the activation of TLR2 and TLR4 expressed on the PMN surface occurs simultaneously with NET formation. To do so, PMN were exposed to *E. bovis* sporozoites for 2 h for the subsequent detection of TLR2, TLR4 and NET components, such as NET-associated histones and extracellular DNA by immunofluorescence microscopy analyses. The expression of TLR2 and TLR4 was observed on the surface of bovine PMNs (Figure 5C,D), confirming our data of significantly enhanced TLR2 and TLR4 expression on *E. bovis*-stimulated PMN obtained by flow cytometry analyses (FACS) (Figure 2A,B). Next, we sought to determine the co-localization of TLR2 and TLR4 (red) with PMN extruded extracellular DNA stained with DAPI (Figure 5A,B, (blue)) with the presence of NET-derived histones, a key feature of NETosis, by using an anti-global histone antibody (Figure 5E,F, (green)). Of note, incubation of PMN with TLR2 and TLR4 antibodies alone does not induce NET formation (Supplementary Figure S2). We visualized PMN undergoing different stages of NET formation, seen as the co-localization of extruded NETs decorated with DNA and global histones (H1, H2A/H2B, H3, H4) together with signaling on TLR2 and TLR4 surface expression, as indicated by yellow arrows (Figure 5G,H). Interestingly, PMN showing initial stages of the NETosis process, seen as a decondensed nucleus positive for DNA and global histones, were also positive for TLR expression (white arrows), indicating that TLR expression concomitant to NETosis occurs soon after encountering *E. bovis*. Nonetheless, some PMN stained positive for TLR expression but not for NETosis (orange arrows), suggesting that simultaneous TLR activation and NET formation are only partial, rather than universal, throughout the experimental exposure. Controls of unstimulated PMN treated with TLR2 and TLR4 antibodies, *E. bovis*-induced NETosis without TLR antibody treatment and positive controls for NETosis (PMA 2 µM and zymosan 1 mg mL^−1^) under the same experimental conditions are found in Supplementary Figures S1–S3.

## 3. Discussion

Since the discovery of PMN-derived extracellular traps (NETs), known in the literature as NETosis, by Brinkman and colleagues, the role of NETs in neutralizing pathogens and stimulating immune responses has been investigated [30]. NET formation in response to bacteria, parasites and viruses has been described and specific mechanisms involved in microbial control continue to be elucidated. More recently, NETs have also been implicated in chronic inflammation and autoimmune diseases, such as rheumatoid arthritis and sepsis, highlighting a detrimental function of NETs [58,59]. It has been suggested that NETs may play a crucial role in inflammatory pathologies associated with several parasitic infections [8,60,61]. 

The apicomplexan protozoan parasite *E. bovis* causes coccidiosis in bovines, a pathogenic disease characterized by severe hemorrhagic diarrhea and dysentery, resulting in weight loss, reduced growth rates and decreased general welfare [50]. Previously, we reported that PMN are important in the host early immune response to *E. bovis* infection [49,51] and others have suggested a protective role of PMN in secondary infection of murine *Eimeria* spp. [62]. In response to *E. bovis,* bovine PMN cast extensive NETs in an active cellular process involving CD11b, ROS production, calcium mobilization, elastase function (NE) and myeloperoxidase (MPO) [7]. Furthermore, *E. bovis*-induced NETs were found to reduce infectivity of sporozoites in vitro [6]. More importantly, this event has also been demonstrated in *E. bovis*-infected intestine in vivo [10]. Here, intestinal PMN were recruited to *E. bovis* infection sites in cattle exhibiting NET release co-localized with NE and histones. Exaggerated NETosis or diminished NET clearance are likely to increase the risk of autoreactivity to NET components and are involved in the pathogenesis of autoimmune and inflammatory disorders, such as glomerulonephritis, chronic lung disease, sepsis, epididymitis and vascular disorders, among others [63,64]. In *E. bovis*-induced NETosis, it is suggested that released NETs might play an important role in very early host innate defence reactions during coccidiosis, thereby contributing to the protection of infected animals and significantly altering the outcome of infection, as some parasitic stages (e.g., sporozoites, merozoites) might be unable to invade host cells [6,7,10].

Activation of PMN in order to generate NETs can be mediated by several means, including hydrogen peroxide production, pro-inflammatory cytokines, such as IFNγ and IL-8, and microbial PAMPs [59,65,66]. PAMPs recognized by TLR2, TLR4, TLR7 and TLR9 have been implicated in recognition of *P. falciparum*, *T. gondii, L. major* and *T. cruzi* [33,41,45,46,67]. Further, TLR2 and TLR4 are required for the complete induction of NETs when *T. cruzi* is cultured with PMN in vitro [46], whereas TLR7 has been described to be critical for the control of *L. major* in mice in vivo. This effect seems to be mediated by ROS and NETosis induction [67]. Bovine PMN constitutively express TLR2 and TLR4 and were previously shown to undergo NETosis in a ROS-dependent manner upon exposure to *E. bovis*; therefore, the present work was carried out to investigate in more detail the role of TLR2 and TLR4 in the recognition and uptake of *E. bovis* sporozoites, pro-inflammatory cytokine production (IL-8) and NET formation. 

Pre-incubation of PMN with directly labeled antibodies to TLR2 and TLR4 prior to *E. bovis* exposure had no impact on phagocytosis of *E. bovis* (Figure 2). Interestingly, though, a strongly enhanced TLR signal was observed on *E. bovis*-exposed PMN, which was not a result of the directly labeled antibodies binding to CSFE-labeled *E. bovis* sporozoites (Figure 1). At this moment in time, we are not able to explain this phenomenon, but assume that it is indeed an increased expression of TLR2/4 as a result of *E. bovis* exposure, and insufficient washing of excess antibodies. To assess whether *E. bovis* indeed signals through TLR2 and TLR4, we next used TLR2- and TLR4/MD2-expressing HEK cells. Only heat-killed *E. bovis* sporozoites or *Eb*Ag induced NF-κB induction, whereas live *E. bovis* sporozoites and media controls did not. This suggested that in this experimental system, live *E. bovis* neither activate TLR2 nor TLR4 (Figure 4). Interestingly, increased TLR2 expression on PMN was significantly greater than for TLR4, which was also seen in regard to a stronger NF-κB activation induced in TLR2-HEK cells compared to TLR4/MD2-HEK cells in response to HK sporozoites and *Eb*Ag. In corroboration with the HEK cell reporter assay results, PMN do not produce increased IL-8 when incubated with live *E. bovis* sporozoites, further indicating that attachment or uptake of these stages by PMN is not reliant on TLR activation (Figure 3). Taken together, these results strongly suggest that the destruction of *E. bovis* sporozoites by heat treatment may expose a TLR2-specific ligand not readily accessible in the viable organism. In addition, live parasites are not fully required for TLR2 or TLR4 recognition of *E. bovis*, as demonstrated by a substantial increase in NF-κB induction via TLR2. 

We observed TLR2- and TLR4-positive staining on the surface of PMN concomitant with NET-derived extracellular DNA and global histones in PMN exposed to *E. bovis* (Figure 5). This co-localization was observed in PMN undergoing NETosis and also those in the early stages of NET formation, although this event was not observed in all cells exposed to *E. bovis*. ROS production has a central role in NETosis, and, interestingly, TLR2 induction of ROS is responsible for NETs in response to *L. major* LPG [67]. Consequently, the release of NETs in response to *E. bovis* is also an active cellular death process involving ROS production [7]. TLR2 expression is associated with PMN-derived extracellular DNA with histones induced by *E. bovis* further suggesting a role for TLR2 in NET induction (Figure 5). The involvement of TLRs in parasite-induced NETosis is complex; MyD88 was found not to be necessary for NET formation in response to *T. gondii* tachyzoites, however, TLR2 and TLR4 are required to recognize GPI extracted from *T. gondii* [42,68]. However, there appears to be an agreement that viable parasite stages are not always required for NETosis to occur. Indeed, soluble antigens (prepared by freeze–thawing similar to *Eb*Ag above) from *T. cruzi* induced TLR2/4-dependent NET formation, highlighting that viable parasites are not always required for the recognition and activation of innate immune leukocytes [46]. In the present study, we showed that by performing immunofluorescence microscopy analysis, live *E. bovis* sporozoites are able to induce TLR2 and TLR4 surface expression on bovine PMNs and this signal occurs simultaneously to NETosis. These events seem relevant to the initiation of stronger host innate immune responses against this parasite orchestrating other leukocyte-derived effector mechanisms [8,20]. However, future research should be performed in order to elucidate additional leukocyte populations as well as pathways involved in *E. bovis*-triggered TLR activation and NET formation. Future molecular analyses of *E. bovis* merozoite-, gametocyte- and oocyst-derived antigens, probably all involved in TLR-derived host innate immune reactions during cattle coccidiosis in vivo, are needed. 

## 4. Materials and Methods

### 4.1. Parasites 

The *E. bovis* (strain H) strain used in the present study was maintained by passages in Holstein–Friesian calves (*n* = 3) for oocyst production as described by [69]. Collection of oocysts, oocyst sporulation and excystation of sporozoites were performed as previously described [69]. Released, free sporozoites were washed three times with sterile phosphate-buffered saline solution (PBS, 400× *g*, 10 min), counted in a Neubauer hemocytometer chamber and thereafter suspended at a final concentration of 2 × 10^6^/sporozoites/mL in cell culture medium RPMI 1640 without phenol red (Gibco, Waltham, MA, USA) containing penicillin/streptomycin (both 100 U/0.1 mg/mL, Gibco) until further experimental use. 

For parasite antigen preparation, *E. bovis* sporozoites were homogenized by repeated freezing followed by sonication (20 kHz, 5 × 15 s pulses) on ice. After centrifugation (11,000× *g*, 4 °C, 20 min), the supernatants were passed through 0.2 µm sterile filters (Merk, Darmstadt, Germany). Protein concentration was determined using the Bradford method [70]. The *E. bovis*-specific antigen (*Eb*Ag) was stored at −80 °C until further use.

For PMN phagocytosis assays, *E. bovis* sporozoites were stained with carboxyfluorescein succinimidyl ester (CFSE) following previously described protocols [9]. Briefly, vital sporozoites where incubated with a 2.5 µM final concentration of carboxyfluorescein succinimidyl ester (CFSE, Invitrogen, Waltham, MA, USA) in sterile PBS (cell culture grade, 0.1 µm filtered, endotoxin tested, Gibco) for 30 min at 37 °C prior to sterile PBS washing by centrifugation (400× *g*, 10 min; three times). CFSE-stained sporozoites were suspended in RPMI 1640 cell culture without phenol red (Gibco) ready for incubation with bovine PMN. 

### 4.2. Isolation of Bovine PMN

Whole blood was collected by venopuncture of the jugular vein from healthy parous female Holstein–Friesian cattle housed at Bolton’s Park Farm, Royal Veterinary College (RVC), London, United Kingdom, using acid citrate dextrose under Home Office license PPL7009059. PMN were isolated by density centrifugation followed by flash lysis as previously described [16]. Briefly, whole blood was diluted with PBS + ethylenediaminetetraacetic acid (EDTA) 0.01% before layering onto Lymphoprep (Biocol, Millipore, Burlington, MA, USA) and centrifugation at 700× *g* for 30 min. The lower layer containing PMN was retained, lysed with distilled water for 40 s and recovered with Hank’s balanced salt solution (HBSS) without phenol red (Gibco) before washing by centrifugation. PMN were counted by a trypan blue (Sigma-Aldrich, Darmstadt, Germany) exclusion test using FastRead^®^ counting chambers (Immunosystems, Torquay, UK) and adjusted to 1 × 10^6^ cells/mL with RPMI 1640 cell medium without phenol red (Gibco). PMN purity was assessed by microscopy after DiffQuick^®^ (Reagena, Toivala, Finland) staining and isolations of at least 90% were used for *E. bovis* stimulation assays. 

### 4.3. Blocking of TLRs and Phagocytosis Assay

All reagents used in these assays were either purchased from specified suppliers as “low LPS, culture grade”, or were tested for LPS in the TLR4 HEK cell system. Isolated PMN (1 × 10^6^ per sample) from three animals were pre-treated with Alexa Fluor 647 (far red spectrum) directly labeled antibodies to TLR2 and TLR4 (AbD Bio-Rad, Watford, Hertfordshire, UK), see Table A1) for 30 min before washing once. PMN were then exposed to CFSE-labeled *E. bovis* sporozoites (CFSE treatment: 2.5 µM, 30 min, 37 °C) at a 1:1 ratio for 2 h. PMN supernatant was collected, clarified and stored for subsequent IL-8 ELISA (AbD Bio-Rad, Watford, Hertfordshire, UK), after which PMN were washed in PBS twice and finally suspended in 400 µL FACSFlow for flow cytometric analysis. Ten thousand events were acquired with a BD FACS Calibur running Cell Quest Pro (BD Biosciences, San Jose, CA, USA) and post-analyzed with FlowJo V10 software (BD Biosciences, San Jose, CA, USA). 

### 4.4. IL-8 ELISA

IL-8 was detected in stimulated PMN cell-free supernatants by capture ELISA as previously described [71]. Antibodies used (Table A1) were mouse anti-sheep IL-8 capture (AbD Serotec), rabbit anti-sheep IL-8 detection (AbD Serotec) and goat anti-rabbit horseradish peroxidase (HRP) detection (Agilent (DAKO), Stockport, Cheshire, UK) alongside recombinant bovine IL-8 (Kingfisher Biotech, Saint Paul, MN, USA) to create a standard curve. Supernatants from stimulated PMN were collected, clarified by centrifugation and stored at −20 °C until the ELISA was performed. Results were visualized by the addition of 3,3′,5,5′-Tetramethylbenzidine (TMB, Sigma-Aldrich, Dorset, UK) for 15 min before stopping the reaction with 0.5 M sulfuric acid (VWR, Poole, Dorset. UK) and plates were read using a Tecan M200 pro plate reader^®^ (Tecan, Reading, Berkshire, UK) and analyzed with GraphPad^®^ Prism software (GraphPad Software, San Diego, CA, USA).

### 4.5. TLR Stimulation Assay

To assess TLR recognition of *E. bovis*-derived antigens (*Eb*Ag), HEK cells expressing bovine TLR2 or bovine TLR4 containing an NF-κB luciferase reporter (NF-κB-luc, Promega, Chilworth, Hampshire, UK) were used [56,72,73]. Briefly, HEK-TLR2 or HEK-TLR4 cells were seeded at a density of 2.5 × 10^5^ cells in 6-well plates using Dulbecco’s Modified Eagle Medium (DMEM Life Technologies, Paisley, Renfrewshire, UK) supplemented with 10% fetal bovine serum (FBS, Life Technologies, Paisley, Renfrewshire, UK) and 1 mg mL^−1^ Geneticin (Life Technologies, Paisley, Renfrewshire, UK). After 24 h, HEK cells were transfected with 250 ng p NF-κB-luc with TurboFectTM (Thermo Scientific, Waltham, MA USA) using the manufacturers’ standard recommendations and allowed to recover for 24 h. For experimental assays, one well of a 6-well plate was split into 6 wells of a 24-well plate and each condition was assayed in triplicate. HEK-TLR2 and HEK-TLR4 cells were exposed to *E. bovis* stimuli: live 2.5 × 10^5^; dead 2.5 × 10^5^ heat killed (60 °C, 30 min) and soluble *Eb*Ag 100 µg for 24 h with TLR2 (1 µg mL^−1^ Pam3CSK4), TLR4 (1 µg mL^−1^ LPS-EK) and NF-κB [100 ng mL^−1^ phorbol 12-myristate 13-acetate (PMA)] controls (all Invivogen, Toulouse, Midi-Pyrenees, France). NF-κB gene activation was determined using the Luciferase Reporter Assay System (Promega Chilworth, Hampshire, UK) following the manufacturer’s instructions. Cell lysates were clarified by centrifugation at 16,000× *g* for 5 min and protein concentration determined by absorbance at 280 nm with a Nanodrop ND-1000 for normalization, as described [74]. 

### 4.6. Induction of NETosis and TLR2 Expression Via Fluorescence Microscopy Analysis

In another set of experiments, *E. bovis*-induced TLR2 and TLR4 expression and NETosis were analyzed via fluorescence microscopy analysis. Here, bovine PMN (*n* = 3; 5 × 10^5^) were exposed to vital *E. bovis* sporozoites at a ratio 1:1 on previously pre-coated poly-l-lysine-treated (Sigma-Aldrich, Darmstadt, Germany) coverslips (2 h, 37 °C) in a plastic 24-well plate (Greiner, Kremsmünster, Austria). Thereafter, the samples were fixed (overnight, 4% paraformaldehyde on ice, Merck), for 20 min at room temperature (RT), washed thrice with PBS and stored at 4 °C until further use. Prior to antibody exposure, samples were washed three times with sterile PBS and blocked with bovine serum albumin (BSA 2%, 30 min, Sigma-Aldrich, Darmstadt, Germany). Treatments with anti-TLR2 and anti-TLR4 antibodies (1 µg mL^−1^, in the dark, detailed in Table A1) were performed for 1 h. Thereafter, samples were carefully washed three times with sterile PBS. For the detection of histones, cells were incubated with a pan-histone antibody detecting H1, H2A/H2B, H3, H4 (MAB3422; Merk, Darmstadt, Germany), diluted 1:200 for 1 h, washed washed twice with sterile PBS and incubated with the secondary antibody (Alexa Fluor 488 goat anti-mouse, Life Technologies, Paisley, Renfrewshire, UK) diluted 1:500 in buffer (PBS 1×, 3% BSA) for 1 h in the dark (for antibody information, see Table A1). Finally, specimens were washed three times with sterile PBS and mounted in ProLong Antifade^®^ containing DAPI (Thermo Fisher Scientific, Waltham, MA, USA) for 24 h in the dark, which were used for the detection of nuclei and NET extracellular DNA. Visualization of TLR expression on the surface of PMN, NET structures based on co-localized extracellular DNA staining and histone-derived signals was achieved by using an inverted Olympus IX81^®^ epifluorescence microscope equipped with an XM10^®^ digital camera (Olympus, Hamburg, Germany). Staining for controls consisting of unstimulated PMN incubated with antibodies to TLR2 and TLR4, *E. bovis*-induced NETosis without TLR antibody treatment and positive controls for NETosis (PMA 2 µM; zymosan 1mg mL^−1^; Sigma-Aldrich, Darmstadt, Germany) can be found in the Supplementary Data. 

### 4.7. Statistical Analysis

Data were analyzed using Microsoft Excel (Microsoft 2013, Redmond, Washington, USA) and GraphPad^®^ Prism. Differences were regarded as significant at a level of *p* ≤ 0.05 (*).

## Data Availability

The data presented in this study are available on request from the corresponding author. The data are not publicly available due to internal policies.

## Supplementary Materials

The following are available online at https://www.mdpi.com/2076-0817/10/2/118/s1. Figure S1: Control of PMN exposed to *E. bovis* without TLR treatment and subsequent NET formation. PMN (*n* = 3; 5 × 10^5^) were exposed to vital *E. bovis* sporozoites (ratio 1:1) on poly-l-lysine-treated coverslips (120 min, 37 °C) and fixed in 4% paraformaldehyde (A) for further antibody exposure (60 min) with an anti-histone H1, H2A/H2B, H3, H4 antibody (C). Coverslips were mounted with ProLong Antifade^®^ containing DAPI (B) which was used for observation of PMN nuclei and NET extracellular DNA. Co-localization of *E. bovis* induced-bovine NET DNA (blue) with NET-derived histones (green) is observed (D). Images were visualized by using an inverted Olympus IX81^®^ epifluorescence microscope equipped with a digital camera (XM10^®^, Olympus, Tokyo, Japan). Scale bar magnitude: 20 µm. Figure S2: Control TLR expression in unstimulated PMN + TLR2/4. PMNs (*n* = 3; 5 × 10^5^) were seeded on poly-l-lysine-treated coverslips (120 min, 37 °C) and fixed in 4% paraformaldehyde for further antibody exposure (60 min) with anti-TLR2 and anti-TLR4 antibodies (red). Thereafter, coverslips were mounted with ProLong Antifade^®^ containing DAPI which was used for observation of PMN nuclei (blue). Images were visualized by using an inverted Olympus IX81^®^ epifluorescence microscope equipped with a digital camera (XM10^®^, Olympus, Tokyo, Janpan). Scale bar magnitude: 20 µm. Figure S3: Control of TLR expression and release of extracellular DNA by PMN on PMN incubated with zymosan (1 mg mL^−1^) and PMA (2 µM). PMN (*n* = 3; 5 × 10^5^) were incubated with zymosan (1 mg mL^−1^) or PMA (2 µM) on poly-l-lysine-treated coverslips (120 min, 37 °C) and fixed in 4% paraformaldehyde for further antibody exposure (60 min) with anti-TLR2 and anti-TLR4 antibodies (red). Thereafter, coverslips were mounted with ProLong Antifade^®^ containing DAPI which was used for observation of PMN nuclei (blue) and PMN-derived extracellular DNA. Images were visualized by using an inverted Olympus IX81^®^ epifluorescence microscope equipped with a digital camera (XM10^®^, Olympus, Tokyo, Japan). Scale bar magnitude: 20 µm.

## Appendix A

**Table A1:** Antibodies.

Antibody	Supplier	Details	Isotype
Alexa Fluor 647 conjugated anti-human TLR4	Novus	NBP2-24773 (clone 76B357.1)	IgG2a
Alexa Fluor 647 conjugated anti-bovine TLR2	Bio-Rad	HCA152A647 (clone AbD12538)	HuCal Fab
Mouse anti-sheep IL-8	Bio-Rad	MCA 1660 (clone 8M6)	IgG2a
Rabbit anti-sheep IL-8	Bio-Rad	AHP425 (polyclonal)	Polyclonal IgG
Goat anti-rabbit HRP	DAKO	P0448	-
Mouse anti-histone	Merck	MAB3422 (clone H11-4)	IgG1
Alexa Fluor 488 goat anti-mouse IgG	Life Technologies	Recombinant polyclonal	IgG
