# Supplementary figures and images for: The Role of TLR2 and TLR4 in Recognition and Uptake of the Apicomplexan Parasite Eimeria bovis and Their Effects on NET Formation
Source: Pathogens. 2021 Jan 24;10(2):118. doi: 10.3390/pathogens10020118 (PMC7912269; doi:10.3390/pathogens10020118)

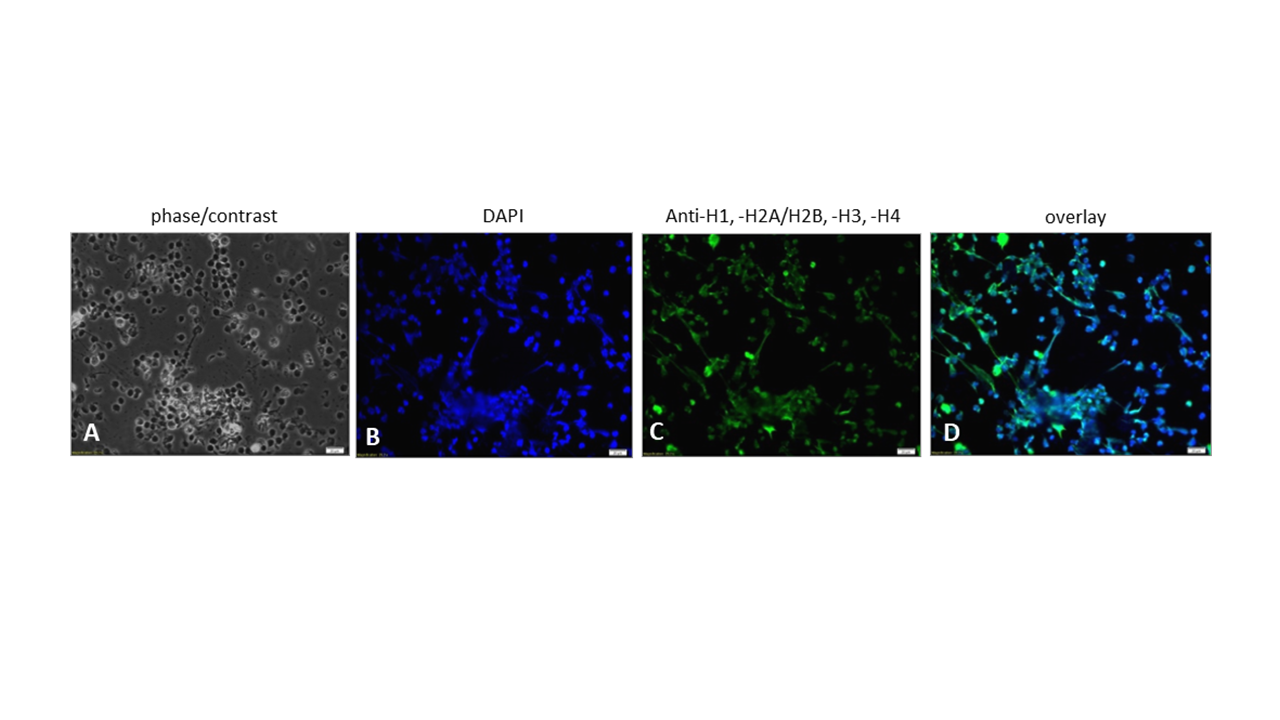

Supplement: Supplementary file 1 [file pathogens-10-00118-s001.zip › Suplemmentary data. Fig. 1.tif]

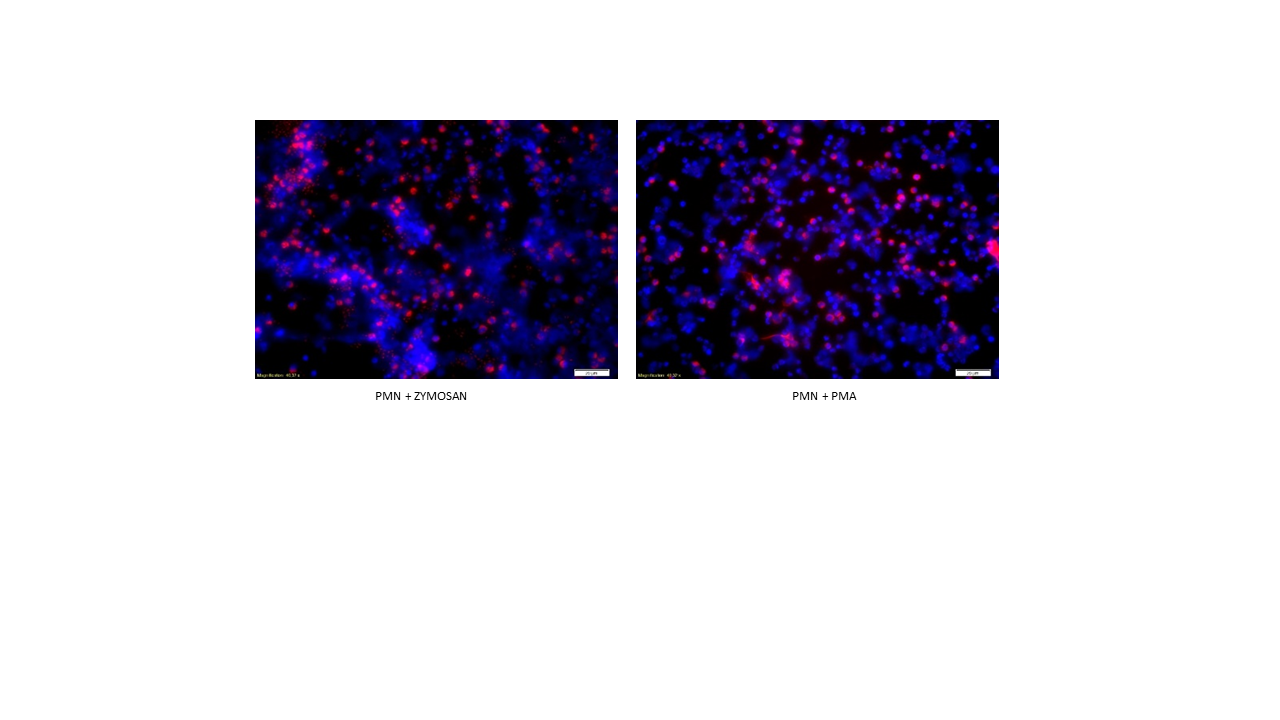

Supplement: Supplementary file 1 [file pathogens-10-00118-s001.zip › Suplemmentary data. Fig. 3.tif]

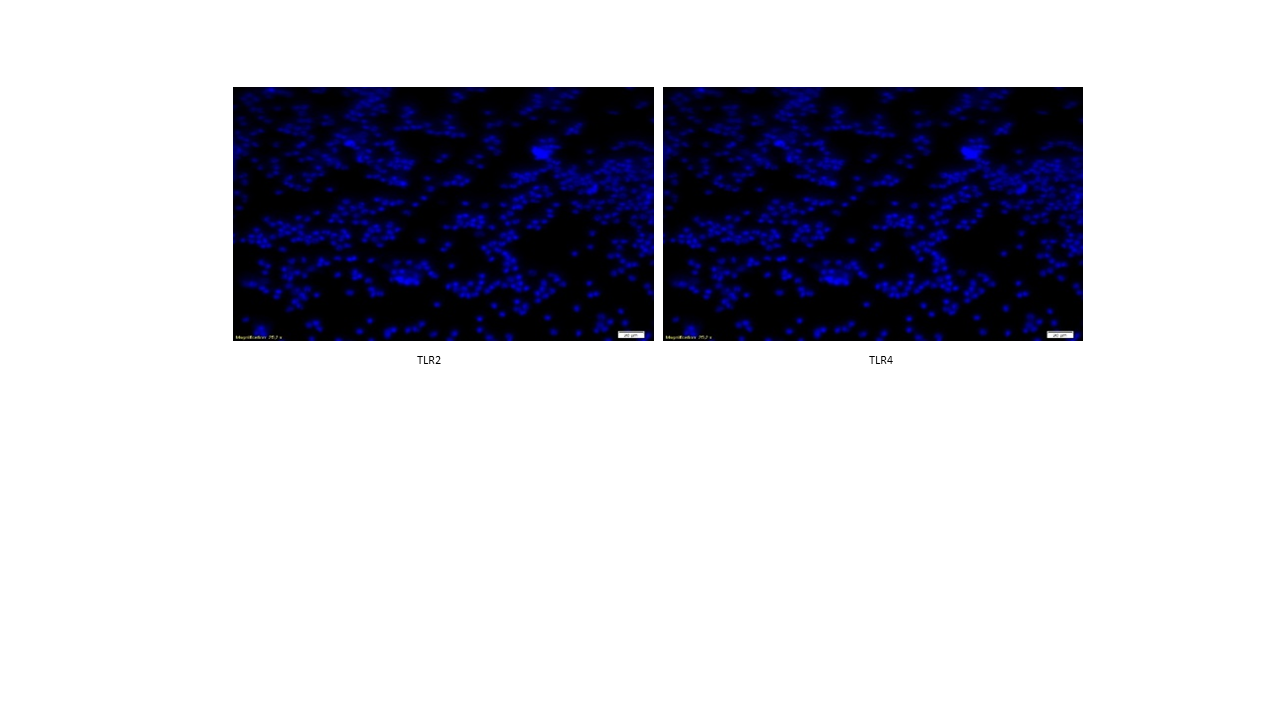

Supplement: Supplementary file 1 [file pathogens-10-00118-s001.zip › Supplementary data. Fig. 2.tif]
